# Summary of the 2015 University of Michigan Sport Concussion Summit

**DOI:** 10.2217/cnc-2016-0007

**Published:** 2016-09-23

**Authors:** Steven P Broglio, Grant Baldwin, Rudy J Castellani, Sara PD Chrisman, Stefan Duma, Brian Hainline, Joanne C Gerstner, Kevin Guskiewicz, Jeffrey Kutcher, Adria Lamba, Michael McCrea, Steven Pachman, Christopher Randolph, Tamara C Valovich McLeod

**Affiliations:** 1 NeuroTrauma Research Laboratory, University of Michigan Injury Center, University of Michigan, Ann Arbor, MI, USA; 2 Centers for Disease Control & Prevention, Atlanta, GA, USA; 3 University of Maryland, Baltimore, MD, USA; 4 Seattle Children's Hospital, University of Washington, Seattle, WA, USA; 5 School of Biomedical Engineering & Sciences, Virginia Tech, Blacksburg, VA, USA; 6 National Collegiate Athletic Association, Indianapolis, IN, USA; 7 School of Journalism, Michigan State University, East Lansing, MI, USA; 8 Department of Exercise & Sport Science, University of North Carolina at Chapel Hill, Chapel Hill, NC, USA; 9 The Sports Neurology Clinic, CORE Institute, Brighton, MI, USA; 10 Georgetown University Law Center, Washington DC, USA; 11 Department of Neurosurgery, Medical College of Wisconsin, Milwaukee, WI, USA; 12 Montgomery McCracken, Philadelphia, PA, USA; 13 Department of Neurology, Loyola University Chicago, Chicago, IL, USA; 14 School of Health Sciences, A.T. Still University, Mesa, AZ, USA

**Keywords:** chronic traumatic encephalopathy, concussion, impact biomechanics, mild traumatic brain injury

## Abstract

Discussions surrounding concussion have made their way into the public sphere over the previous decade with media attention and coverage of the injury fueling public debate. These conversations have devolved into discussions on banning contact and collision sports and raised legal questions surrounding injury management. Questions raised about concussion eclipse what science can answer, but the University of Michigan Injury Center (MI, USA) hosted a Concussion Summit in September 2015 as a means to condense, solidify and disseminate what is currently known on the topic. Areas for discussion included concussion incidence and prevention, diagnosis and management, legislation and education, legal and social aspects and future directions. A summary of those presentations are included within.

## Background

Interest in concussions has risen over the previous decade, with a particular focus on improving injury identification, diagnosis, management and understanding the potential for long-term consequences. As awareness and knowledge in the general population has increased, researchers have sought to answer many unanswered questions posited in the literature and by the media. The result has been a nearly fivefold increase in sport concussion research in the previous 10 years compared with the 10 years prior. With a rapid increase in knowledge fueled by research efforts, information dissemination is critical. To better publicize information from this rapidly evolving area of research, the University of Michigan Injury Center (MI, USA) hosted a Sport Concussion Summit in September 2015. Speakers from across the country were invited to address five broad topics targeting a wide array of stake holders (i.e., researchers, clinicians, administrators, athletes, coaches, parents, attorneys and media). Although the literature in this area is changing rapidly, within this manuscript is a summary of those presentations that highlighted relevant works and expert opinion on each topic.

## Concussion incidence & prevention

The foundation of injury research lies in understanding true injury incidence. Recent media reports about the dangers of head trauma in sport have led the assumption that sport concussion incidence has risen dramatically and there is now a ‘concussion crisis’. In reality, the increased reported concussion incidence can be attributed to both concussion legislation in all 50 states and media influence [1]. While the specifics vary from state to state, each state law generally requires concussion education and improved concussion diagnosis and management for youth and adolescent athletes. Therefore, increased awareness and improved management is most likely capturing previously undiagnosed concussions in high school athletes. This is highlighted at the collegiate level, whereby concussion laws do not address intercollegiate athletics and concussion incidence rates at that level have remained consistent over the last 10 years, with only lacrosse and football showing subtle increases during this time [2]. Concussion incidence in professional football increased beginning from 2010 to 2012, but a subtle decrease was observed in 2013–2014 with significantly fewer game-related concussions reported. These shifts coincide with the National Football League's (NFL's) policy and rule changes directed at improving player safety. It is thus believed that concussion education and legislation, coupled with better assessment tools, is likely leading to increase concussion diagnoses, although this does not necessarily translate to an increase in the true number of concussions occurring on our playing fields.

As awareness of sport concussions grows, so too does research targeted at reducing injury risk. For the past 60 years, researchers have performed experiments aimed at better understanding brain injury, under the premise that reducing forces on the human body will reduce risk of injury [3]. These started in the 1950s with a small series of tests on human cadavers that served as the foundation for understanding human tolerance to head impact. Injury metrics derived from this work, such as severity index (SI) and head injury criterion (HIC), have been used extensively to design safer automobiles, military protection and athletic helmets. Through the 1970s and 1980s, most research focused on evaluating linear and rotational acceleration's contribution to brain injury through primate experiments. This research illustrated that brain injury is not a function of linear or rotational acceleration in isolation of each other, but rather a combined effect of linear and rotational loading. In the 1990s and early 2000s, the NFL reconstructed concussive football impacts with instrumented test dummies [4] and illustrated that linear and rotational acceleration values are both associated with concussion. Starting in 2003, acceleration data from over two million head impact events have been directly recorded from youth, middle school, high school and collegiate football players [5,6]. These data have allowed for the development of injury risk functions that can be applied toward designing products to reduce concussion risk [7]. Interestingly, over 60 years of research utilizing very different experimental methodologies demonstrate the same trend: lowering both linear and rotational accelerations lowers brain injury risk. This combined research provides the foundation for helmet testing in order to evaluate the relative differences in biomechanical performance. Helmets are not the solution to preventing all concussions, but helmets that better modulate impact energies, and therefore lower both linear and rotational acceleration, may reduce concussion risk [8]. The Virginia Tech Helmet Ratings analyze both linear and rotational acceleration values for all sports as a mechanism to help improve helmet design and inform consumers of the relative performance of different models [9,10].

## Concussion diagnosis & management

The diagnosis and management of concussion requires a critical and skillful examination of the human nervous system combined with a comprehensive approach to patient management by appropriately trained medical professionals. A highly attuned skillset is necessary given the ease by which concussion symptoms can be hidden by the athlete and the intense scrutiny placed on these injuries. Because of the complexity in how concussive injuries present, many evaluative tools (i.e., motor control and cognitive functioning assessments) have been developed to aid the clinician in both the field and clinic. Unfortunately, these tools often oversimplify diagnostic schema and nonsport-specific approaches to injury evaluation, resulting in frequent misuse. As each sport has a unique set of brain injury risks and challenges for clinicians, sport specific approaches should be emphasized [11], rather than overgeneralizing a single best approach to all concussions.

The diagnosis of concussion is quite often steeped in a degree of uncertainty and focusing on the concussion alone often leaves a large portion of a patient's overall brain health unaddressed. Whether explicitly stated or not, clinicians may categorize these injuries as ‘definite’, ‘probable’ or ‘possible’ at the time of injury. Framing concussions in this fashion helps provide a level of clarity to the overall clinical picture and may lead to more efficient management. To develop a clear clinical picture, each patient with a suspected or diagnosed concussion deserves a thorough evaluation that includes their medical history, including previous exposure to contact, estimation of their future exposure risk and delineation of potential negative effects on their overall brain function [12].

To supplement the individualized approach to concussion diagnosis and management, researchers have pursued a single biomarker that is both diagnostic and prognostic of clinical outcomes. Scientific advancements over the past 30 years, however, suggest that such a single biomarker does not likely exist. Rather, it has become clear that multiple predictors from biological, environmental, genetic, social and psychological domains operate jointly to affect a range of possible outcomes after concussion. In addition, recovery and outcome can no longer be viewed as a unidimensional construct, but one constituted by multiple vectors of recovery (e.g., cognitive and neurobehavioral function, psychological health, life function and quality).

The combination of predictor and outcome variables creates a complex, neurobiopsychosocial model of mild traumatic brain injury (mTBI) [13]. The neurobiopsychosocial model suggests that to understand and predict outcome after concussion requires a broad matrix of predictor domains that incorporate pre-injury function (e.g., cognitive, behavioral and psychosocial function, genotype), injury specifics and context (e.g., severity, frequency, mechanism), immediate post-injury events (e.g., acute characteristics, diagnosis, treatment), and intervening life events (e.g. life stressors). Neuroimaging and blood biomarkers are of primary interest in this arena [14]. In parallel, a rich, multidimensional approach to outcome measurement (e.g., neurobiological, cognition, psychological health, quality of life, vocational/life function) is critical to capture the full outcome spectrum.

Modern research efforts are leveraging the power of the sports concussion research model to advance the clinical and basic science of mTBI [15]. One such initiative is the Concussion Assessment, Research and Education (CARE) Consortium, a large-scale effort cosponsored by the National Collegiate Athletic Association (NCAA) and Department of Defense, geared toward informing the true natural history of recovery after concussion in athletes. The CARE and other parallel efforts integrate biomechanical, clinical, neuroimaging, neurobiological and genetic markers of injury to advance our understanding of neurophysiological effects and recovery after concussion.

The CARE and similar studies offer the promise of informing the lay population and clinicians on the nature and frequency of various short- and long-term risks associated with concussion. To date, research findings suggest that the short-term recovery from concussion is typically rapid and complete [16]. In instances when rapid recovery does not occur, some have theorized that long-lasting ‘post-concussion’ symptoms in nonsports settings are due to psychological factors, and these same factors may play a role in the recovery for some athletes as well [17,18]. Our knowledge surrounding the long-term effects of concussion, or exposure to repetitive head trauma in sports, and an elevated risk of late-life neurodegenerative disease, including the existence of a unique clinical syndrome (e.g., chronic traumatic encephalopathy [CTE]) in retired athletes [19,20] is mixed. Indeed, no prospective, case–control or cohort studies exist that indicate an association between concussion and altered p-tau at autopsy. Conclusions made to date linking repeated head impacts and CTE are based on case studies, and while alluring, lack prevalence data and control groups. Moreover, others have shown that phosphorylated tau accumulations occur throughout human life with no adverse consequences. In addition, the clinical features described with chronic repetitive concussion vary widely and are impossible to separate from other conditions during life, such as depression and known neurodegenerative diseases [21–23].

Conversely, consensus has been developed on how to identify CTE postmortem; with recent literature suggesting that the accumulation of phosphorylated tau occurs in a particular distribution in contact sport athletes [20]. The paradigm for disease pathogenesis, which appears widely accepted, consists of concussions or subconcussive forces acting on brain parenchyma to produce a deleterious neuroinflammatory cascade which, through experimental models, has been suggested to involve tau templating and trans-synaptic neurotoxicity [21]. This occurs especially in the sulcal depths and perivascular areas of the frontal, temporal and insular cortices and may be one consequence of concussion, given the assumption that athletes have increased exposure to pathophysiologically significant head impacts [24]. The distribution of tau proteinopathy has been associated with various clinical signs, including headache, memory complaints, impulsivity, explosive anger and heightened suicidality [24]. A progressive quality to the condition reminiscent of neurodegenerative diseases, such as Alzheimer's disease, has also been suggested as the common outcome with the passage of time (as long as decades) [24]; but much additional research is needed to determine if this unique neuropathology is directly associated with clinical features downstream of repetitive mild neurotrauma.

## Bringing about change: legislation & education

Concussion legislation originated with the Zackery Lystedt Law [25,26], passed in Washington State in 2009. Zackery Lystedt was a middle school football player who was removed from play for a ‘ding’, but then went back in the game, sustained another hit, collapsed and was later found to have a subdural hematoma. The Lystedt Law was designed to prevent youth from playing with concussive symptoms and thus risking a similar injury, and includes three criteria: any athlete with suspected concussion must be removed from play; the athlete cannot return without written clearance by a healthcare professional; and coaches, parents and youth must receive concussion education. The law also provides liability coverage to schools that follow these guidelines. Six years following the passage of the first concussion law, all states have enacted similar legislation [27,28], with some variability regarding specific providers who can clear athletes, type of concussion education required, inclusion of liability protection and ages covered. A few states have extended these laws to mandate decreased full-contact practices or return to learn protocols. Preliminary studies suggest increased diagnosis and healthcare utilization for concussion following the passage of concussion laws [1,29], but continued high rates of youth playing with concussive symptoms [30–32]. This suggests different approaches may be necessary to prevent youth from playing with concussive symptoms [30–32]. Several studies have highlighted barriers to concussive symptom reporting [33–40], and this research may result in new recommendations regarding targeted education for youth, parents and coaches.

Concussion education centers on the recognition of and response to concussion and is important for all stakeholders to ensure appropriate actions are taken to manage suspected injuries. In some states, the education of various stakeholders is mandated by law [27,41] (detailed above) and numerous concussion education curricula are available for different stakeholder groups. The inclusion of concussion education into many state laws and association policies results from studies that have found a lack of awareness and understanding regarding concussion among athletes [39,42–47], coaches [48–50], parents [51–53] and even medical providers [54–59] in addition to failure on the part of athletes to report symptoms of concussion [39,44,60,61]. While the reasons for nondisclosure vary, they often include lack of awareness that the injury could be a concussion or that the injury is serious enough to warrant disclosure.

While understanding stakeholder knowledge of concussion is important in developing educational tools, educational efforts need to focus on changing attitudes and ultimately improving reporting behaviors [38,40]. More recent concussion knowledge studies seem to indicate improved knowledge [62–65] does not always result in altering behavior to increase reporting [37,39,40,66]. These studies have used public health approaches [38,40] that have generated conflicting results. One study [67] noted a significant increase in perceived unsafe reporting behaviors (e.g., more likely to continue play) after watching an educational video. The other [39] demonstrated increased knowledge correlated to increased prevalence of concussion reporting and better concussion attitude scores resulting in fewer athletes indicating they participated while symptomatic.

The use of knowledge translation (KT) may assist in turning knowledge improvements into actionable items that may better impact concussion management. KT is defined as “the exchange, synthesis, and ethically sound application of knowledge within a complex system of interactions among researchers and users” [68]. With respect to concussion, the goal is to determine whether concussion education can modify reporting intention and behavior to allow concussions to be recognized and managed appropriately. Effective KT includes developing educational pieces specific to target audiences and measuring the outcomes of interest [69]. Therefore, education cannot be a one-size-fits-all approach. The message must be individualized for different stakeholder groups and use different delivery methods, including internet, in-person presentations, video games or social media.

## Legal & social aspects of sport concussion

With increased scrutiny placed on the diagnosis and management of concussive injuries, more and more lawsuits are alleging the failure of schools, healthcare providers and athletic associations to meet the appropriate standard of care are being filed. The popularity of these actions is attributable not only to the increasing awareness of concussive injuries, but also because plaintiffs’ attorneys are now targeting so-called ‘concussion’ cases. In what is becoming a trend for athletic associations, schools, coaches and healthcare providers who treat athletes, today's reality is that following a concussion in the sporting context with poor outcomes, the first question becomes who – other than the injured athlete – is responsible for that outcome. That question quickly then becomes who must ultimately pay the injured athlete or the surviving family to compensate for that injury.

In the legal defense of medical professionals, schools and other entities in concussion cases, a litany of questions can be anticipated and often include (but not limited to): was the athlete properly educated about the signs and symptoms of concussions? Should the athletic trainer have conducted additional testing on an injured athlete prior to returning the athlete to game play? Was the athletic trainer working under the supervision of a physician? Was the school's concussion policy adequate? Should preseason neuropsychological baseline testing have been implemented as part of the policy? Did the coach unduly influence the athletic trainer's best judgment?

Lawsuits against medical providers and their employers are partly driven by increased awareness of concussions that has permeated the US media, with increasing amounts of daily reporting on the subject, both within and outside the sports sections. Notably, it is now common for the term ‘concussion’ to be heard in game reports, practice stories and even feature stories in a frequency not seen before 2006.

Indeed, the intensity of focus on concussions has been keyed by a few factors: in 2007, *The New York Times* sports department began an award-winning series of stories on concussions, football players struggling with post-concussive issues, leading to heightened national attention and Congressional hearings; high-profile NFL stars committing suicide and the ensuing coverage, especially the deaths of safety Dave Duerson and linebacker Junior Seau; lawsuits filed against the NFL, leading to the specialty of sports concussion litigation; the fracturing of media sources from traditional sources to website and social media channels – which now deliver articles and video content; the acute competition for audience, leading to ever increasing sensationalism and a rush to publication to be first; a general lack of understanding of concussion by mainstream journalists – and a lack of consistent communication about issues by practicing neurological professionals. Each of these factors has brought concussion into the public domain and placed pressure on sports leagues and legislative bodies to examine their policies and procedures to increase the safety of participation for their players. These same factors also appear to have led to increased uneasiness among the public about sports participation and risk of injury.

## Future perspective

The increased discussion of concussions and sports within the mainstream media spheres has increased awareness to where it is now a public health concern in the USA [70–73]. Seminal research in this field has provided a foundation for understanding some fundamental aspects of recovery following concussion [16,74], but these studies were male-dominated and focused on football and ice hockey. Furthermore, clinical studies of concussion focus on clinical return to baseline and do not address neurobiological recovery. Indeed, there is a paucity of data correlating clinical recovery and neurobiological recovery following concussion. In addition to these barriers, current concussion knowledge has not been associated with a cultural shift in reporting concussion and assuring that concussed athletes are not placed in an environment in which return-to-play is influenced by stakeholders other than treating clinicians [36,75,76].

The 2013, Institute of Medicine report on sports-related concussions [77] made two focused recommendations around these shortcomings. The first was a call for the Centers for Disease Control and Prevention (CDC) to improve the tracking and monitoring of youth sports and recreation-related concussions. Second, CDC was asked to develop, implement and evaluate the effectiveness of large-scale efforts to increase knowledge about concussions and change the culture surrounding concussions. More than 10 years ago, CDC introduced the Heads-Up initiative and the *Heads Up: Opportunities to Reshape the Culture around Concussion*, which provides a snapshot of current research on concussion knowledge and awareness and how concussion affects young athletes’ attitudes and behaviors both on and off the playing field [78]. This information on the implementation and effectiveness of the Heads-Up materials will extend the scope of the program to help change social norms.

To address other focus areas, *CDC Injury Center Research Priorities* outlined a set of targeted research priorities for the next 3–5 years that focuses on addressing strategic gaps in the field. Addressing these gaps is critical to reducing the burden of concussions among youth [79]. As a result, the CDC is developing pediatric guidelines for the appropriate diagnosis and management of children and teens with mild TBI. A collaborative effort between the CDC and leading concussion experts resulted in a systematic and graded process to evaluate the state of the science with publication of the guidelines scheduled for 2016.

In a similar vein, the NCAA partnered with the federal government and numerous medical organizations to promote and develop key research initiatives and concussion guidelines. Most prominently, the NCAA-Department of Defense Grand Alliance [80] is a joint venture that consists of two projects: The CARE Consortium is the largest prospective, longitudinal clinical study ever conducted in the history or concussion. The purpose is to define the natural history of concussion and neurobiological recovery following concussion. The Mind Matters Challenge consists of a short-term educational challenge and a long-term research challenge whose goal is to create a paradigm shift in perceived norms and the culture of concussion for all stakeholders. In addition, partnerships with key medical organizations have created and disseminated three important concussion documents [81]: (1) Independent Medical Care for College Student-Athletes; (2) Year-Round Football Practice Contact; (3) Concussion Diagnosis and Management. Collectively, these works have begun to better inform medical care and shift the culture of sport concussion at the collegiate level.. Substantially more work is needed, however, among young sport athletes.

## Conclusion

The science behind sport concussion is rapidly evolving and this document aims to consolidate and share the information disseminated by the invited speakers at the University of Michigan Injury Center Sport Concussion Summit. The purpose of the meeting was to provide a combination of research and expert opinion to deliver a balanced view of concussion to researchers, clinicians, administrators, athletes, coaches and parents. Given the many unknowns and the rapid increase in research in this area it is likely that our understanding of the topic will be a shifting target, but the general principles outlined here will likely remain.

**Executive summary**

**Presentations given during this summit indicated**

- True concussion incidence has likely not changed, but injury reporting has improved.
- The concussion diagnosis combines the art of clinical practice with the science supporting broadly evaluated assessments.
- Concussion legislation and education efforts have been positive in improving both injury reporting and management.
- Medical practitioners should strive to remain up-to-date on best practices and adhere to them as closely as possible.
- Prospective investigations are needed to elucidate the long term effects of concussion and repeated head impacts.
